# Identify the Early Predictor of Mortality in Patients with Acute Paraquat Poisoning

**DOI:** 10.1155/2020/8894180

**Published:** 2020-12-31

**Authors:** Jun Wang, Xinrui Jiang, Geng Lu, Jiawei Zhou, Jian Kang, Jin-song Zhang

**Affiliations:** ^1^Department of Emergency, The First Affiliated Hospital of Nanjing Medical University, 300 Guangzhou Road, Nanjing, Jiangsu 210029, China; ^2^Department of Emergency, Nanjing Drum Tower Hospital, Medical School of Nanjing University, Nanjing, China

## Abstract

**Background:**

Paraquat is a widely used nonselective and fast-acting contact herbicide worldwide. This study identified the early predictor of mortality in patients with acute paraquat poisoning.

**Methods:**

Twenty-nine patients with acute paraquat poisoning admitted at Nanjing Drum Tower Hospital from January 2018 to August 2020 were included in this study. The early predictor of mortality in patients with acute paraquat poisoning based on the blood tests was identified by correlation, logistic regression, and receiver operating characteristic (ROC) analyses.

**Result:**

15 of the 29 patients died after poisoning. Compared to the survivors, the neutrophilic granulocyte ratio, leukocyte count, ALB, and Crea of the nonsurvivors were significantly higher with *p* value < 0.05, while the lymphocyte ratio and eGFR(MDRD) of the nonsurvivors were remarkably lower with *p* value < 0.01. Moreover, the neutrophil-to-lymphocyte ratio (NLR) was remarkably upregulated in the nonsurvivors. The area under the ROC curve (AUC) of the neutrophilic granulocyte ratio, lymphocyte ratio, leukocyte count, ALB, Crea, eGFR(MDRD), and NLR to predict the mortality in patients with acute paraquat poisoning was 0.8905 (95% CI: 0.7589-1.022), 0.8643 (95% CI: 0.7244-1.004), 0.8500 (95% CI: 0.7133-0.9867), 0.7286 (95% CI: 0.5338-0.9233), 0.8167 (95% CI: 0.6620-0.9713), 0.8714 (95% CI: 0.7330-1.010), and 0.8667 (95% CI: 0.7277-1.006), respectively. More interestingly, we also evaluated the diagnostic values of the different combinations of six blood test biomarkers by logistic regression analysis. According to the results of the logistic regression analysis, the AUCs for the combination of the neutrophilic granulocyte ratio, leukocyte count, and eGFR(MDRD) were the largest with 0.986 (95% CI: 0.952-1), and the sensitivity and specificity were 100% and 100%.

**Conclusion:**

This study demonstrated that the combination of the neutrophilic granulocyte ratio, leukocyte count, and eGFR(MDRD) could serve as an ideal early predictor of mortality in patients with acute paraquat poisoning. However, further research is needed to draw a clear conclusion.

## 1. Introduction

Paraquat is a widely used nonselective and fast-acting contact herbicide worldwide [1, 2]. However, paraquat could also be absorbed by the human body through the skin, respiratory tract, and digestive tract, resulting in multiple system toxicity [3, 4]. Oral administration is the main route of acute paraquat poisoning since paraquat is nonvolatile and non-fat-soluble. The lethal dose for adults is very low, about 20 mg/kg. The plasma concentration of paraquat reached the peak at 2 hours after oral intake and gradually decreased after 15-20 hours later [3]. Paraquat can be rapidly distributed to the lung, kidney, liver, muscle, and other tissues after oral intake, which can cause injury to these organs. However, the lung was the main target organ of paraquat, and its concentration in the lung was 10 to 90 times higher than that in the plasma. Paraquat could hardly bind to the plasma protein and could not be reabsorbed by renal tubules. If the renal function of the patients was normal, 90% of paraquat absorbed into the blood could be excreted in urine within 12-24 hours. The remaining 10% would be released into the blood again after being absorbed by the tissue and then discharged through the kidney, and the part not discharged could be reabsorbed by the tissue again, thus forming a vicious cycle, causing continuous damage to the human body, while the clearance rate of paraquat could be reduced by 10-20 times if the renal function is damaged [5].

In pesticide poisoning, paraquat poisoning is second only to organophosphorus poisoning. However, paraquat poisoning causes the largest number of deaths, and its mortality rate is as high as 60-80%. At present, there is no specific antidote for the treatment of paraquat poisoning. For patients with severe poisoning, it is difficult to improve the prognosis even with various treatment methods, while for some patients with mild poisoning, even if no treatment is used, the prognosis might be good. Therefore, the early prediction of the severity of acute paraquat poisoning is of great help to guide reasonable treatment. However, there is still no unified standard for the prognosis of acute paraquat poisoning despite the fact that the clinical basic research on paraquat poisoning has been carried out for many years. The concentration of paraquat in blood or urine was usually used to evaluate the prognosis of acute paraquat poisoning, but it has not been widely used in most hospitals because it needs extremely expensive, technical, and accurate equipment [3, 6, 7]. Only some large medical centers could detect the concentration of paraquat in blood and urine, especially in developing countries. In addition, the dose of paraquat by oral intake is easily affected by the patient's educational level, description, vomiting, timely gastric lavage, and other differences. It is often difficult for doctors to judge the progress of the disease according to the dose of paraquat poisoning. Therefore, it is urgent to find simple, cheap, and effective biomarkers to evaluate the prognosis of patients with acute paraquat poisoning.

A blood test is a prerequisite for the hospital admission of patients. At present, almost all hospitals have the conditions to carry out blood examinations. Therefore, in this study, we tried to identify an early predictor of mortality in patients with acute paraquat poisoning based on the blood test.

## 2. Method and Materials

### 2.1. Patients

29 patients admitted to Nanjing Drum Tower Hospital with acute paraquat poisoning from January 2018 to August 2020 were included in this study (Table s1). The study was conducted in accordance with the ethical guidelines of the 1975 Declaration of Helsinki and was approved by the Ethics Committee of Nanjing Drum Tower Hospital.

### 2.2. Data Collection

Patient characteristics and clinical features, including age, gender, time from poisoning to treatment (h), and toxic dose (mL), were obtained from the medical record of the patients admitted to Nanjing Drum Tower Hospital with acute paraquat poisoning. The blood test including the blood routine test and blood biochemistry test at admission was obtained before treatment.

### 2.3. Statistical Analysis

The SPSS software version 13.0 (SPSS Inc., Chicago, IL) was performed to the analyses. The independent-sample *t*-test was used to evaluate measurement data, and data were presented as mean ± standard deviation; otherwise, two independent-sample nonparametric tests were performed, and data were presented as median ± interquartile range. Factors with a value of *p* < 0.05 in the univariate analysis were further analyzed using a multivariate Cox proportional hazard model. Receiver operating characteristic (ROC) analysis was used to study the discriminatory power of serum amylase with respect to mortality. Two-tailed *p* < 0.05 was considered statistically significant for all statistical procedures.

## 3. Results

### 3.1. Patient Characteristics

**Figure 1:**
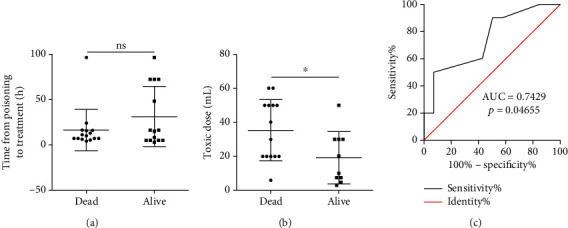
Time from poisoning to treatment (a), toxic dose (b), and receiver operating characteristic curves (c) of toxic dose for the survivors (alive) and nonsurvivors (dead) with acute paraquat poisoning.

From January 2018 to August 2020, 29 patients were admitted to Nanjing Drum Tower Hospital because of acute paraquat poisoning and included in this study (Table 1). Among the 29 patients, mortality was 51.72% (15 patients died). The average age of survivors (alive) and nonsurvivors (dead) was 40.9 ± 15.6 and 46.2 ± 20.8, respectively. There were no significant differences in sex and time from poisoning to treatment between survivors (alive) and nonsurvivors (dead) (Table 1 and Figure 1(a)).

### 3.2. Association between Concentration of Paraquat and Mortality

Since the concentration of paraquat was usually used to evaluate the prognosis of acute paraquat poisoning, we also investigated the concentration of paraquat and mortality. As previously reported [3, 6], the concentration of paraquat was slightly positively correlated with the mortality of acute paraquat poisoning (*p* < 0.05) (Table 1 and Figure 1(b)). However, the area under the ROC curve of plasma paraquat concentrations was just 0.7429 with *p* = 0.04655 (Table 1 and Figure 1(c)). Moreover, we also found that the dose of paraquat by oral intake is hard to accurately measure because of various reasons in several patients (Table 1). For example, several patients were poisoned because of eating vegetables or fruits sprayed with paraquat (Table s1). In these cases, it is almost impossible to assess how much paraquat the patient had taken. The concentration of paraquat in the blood and urea is useful in the prediction for the prognosis of acute paraquat poisoning, but the detection of the concentration of paraquat is extremely expensive, needs technical and accurate equipment, and takes a long time to detect. Also, the concentration of paraquat cannot be detected in time. Therefore, it is necessary to find novel biomarkers to evaluate the prognosis of patients with acute paraquat poisoning.

### 3.3. Blood Test and Prognosis

**Figure 2:**
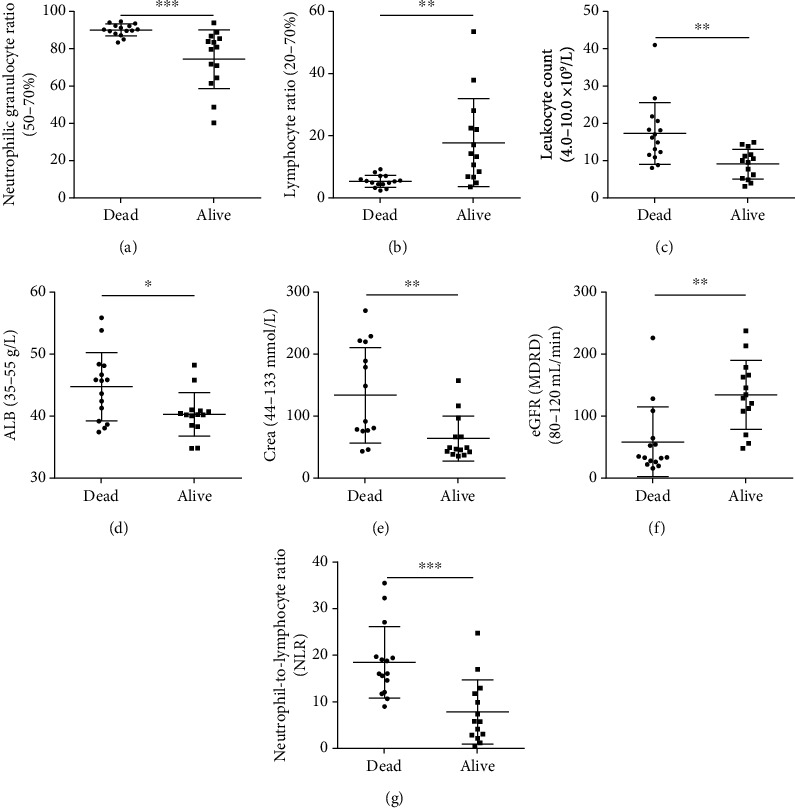
The level of the neutrophilic granulocyte ratio (a), lymphocyte ratio (b), leukocyte count (c), ALB (d), Crea (e), eGFR(MDRD) (f), and NLR (g) for the survivors (alive) and nonsurvivors (dead) with acute paraquat poisoning at admission.

Patient blood test values including blood routine and biochemistry at admission were examined before any treatment, and we found that the neutrophilic granulocyte ratio, leukocyte count, albumin (ALB), Crea, and neutrophil-to-lymphocyte ratio (NLR) of the nonsurvivors were significantly higher than those of the survivors with *p* value < 0.05, while the lymphocyte ratio and eGFR(MDRD) of the nonsurvivors were remarkably lower with *p* value < 0.01 (Figure 2). These results indicated that the neutrophilic granulocyte ratio, leukocyte count, ALB, Crea, lymphocyte ratio, NLR, and eGFR(MDRD) might serve as the early predictor of mortality in patients with acute paraquat poisoning.

### 3.4. ROC Curve Analysis for Mortality

**Figure 3:**
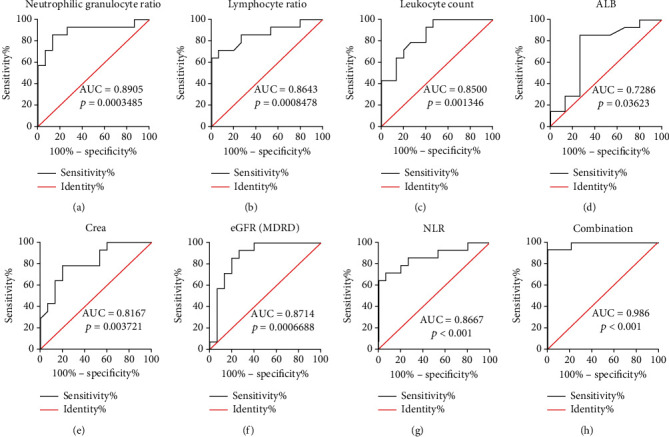
Receiver operating characteristic curves of the neutrophilic granulocyte ratio (a), lymphocyte ratio (b), leukocyte count (c), ALB (d), Crea (e), eGFR(MDRD) (f), NLR (g), and combination of the neutrophilic granulocyte ratio, leukocyte count, and eGFR(MDRD) (h).

Subsequently, we performed the ROC curve analysis to investigate the diagnostic value of the six blood test biomarkers for mortality. The AUCs of these seven blood test biomarkers ranged from 0.7286 to 0.8905 (Figures 3(a)–3(g)). In particular, the neutrophilic granulocyte ratio presented the largest AUC 0.8905 (95% CI: 0.7589-1.022), followed by eGFR(MDRD) 0.8714 (95% CI: 0.7330-1.010). The sensitivity and specificity of the neutrophilic granulocyte ratio were 85.71 and 86.67, respectively. For eGFR(MDRD), the sensitivity and specificity were 92.86% and 73.33%. In addition, the diagnostic values of the different combinations of the seven blood test biomarkers were evaluated by logistic regression analysis. The AUCs for the combination of the neutrophilic granulocyte ratio, leukocyte count, and eGFR(MDRD) were the largest with 0.986 (95% CI: 0.952-1) (Figure 3(h)), and the sensitivity and specificity were 100% and 100%. According to the results of the above logistic regression, we got the risk score (Logit(*p*)) for mortality based on the neutrophilic granulocyte ratio, leukocyte count, and eGFR(MDRD) (Table 2).

(1) $$\begin{array}{c} \text{Logit}\left(p\right)=0.678*\text{neutrophilic granulocyte ratio}+0.484*\text{leukocyte count}-0.034*\text{eGFR}\left(\text{MDRD}\right)-62.54. \end{array}$$

These results suggest that the combination of the neutrophilic granulocyte ratio, leukocyte count, and eGFR(MDRD) was the ideal early predictor of mortality in patients with acute paraquat poisoning.

## 4. Discussion

To our knowledge, this study is the first to identify the early predictor of mortality in patients with acute paraquat poisoning based on the blood test. Our results showed that the combination of the neutrophilic granulocyte ratio, leukocyte count, and eGFR(MDRD) could serve as an ideal early predictor of mortality in patients with acute paraquat poisoning.

The concentration of paraquat in the plasma and urine has been regarded as an effective parameter for the prediction of clinical outcome [2, 3, 6, 8]. However, there are too many limitations for the concentration of paraquat in the plasma and urine to be widely used. Firstly, it required extremely expensive, technical, and accurate equipment to determine the concentration of paraquat in the plasma and urine [3, 6, 7]. Since acute paraquat poisoning is a rare disease, only some large medical centers could be equipped with this equipment and have the ability to detect the concentration of paraquat. In addition, most of the patients could not clearly describe the dose of paraquat by oral intake because of the patient's educational level, vomiting, timely gastric lavage, different brands of pesticides with different doses of paraquat, and other differences. Thus, the dose of paraquat by oral intake was hard to use in the prediction of the prognosis of patients with acute paraquat poisoning in the actual situation. However, it is very important to predict the prognosis of patients at admission because different patients need different treatments. For patients with severe poisoning, there is almost no way to improve the prognosis, but for some patients with mild poisoning, patients can be cured. Therefore, a simple, cheap, and effective method to evaluate the prognosis of patients with acute paraquat poisoning is urgently needed.

At admission, almost all the patients are required to do a blood test to help doctors find out the cause and treatment method. In this study, we explored the possibility of using the blood test biomarker to predict the prognosis of patients with acute paraquat poisoning. By correlation, logistic regression, and receiver operating characteristic analyses, we established a math model to evaluate the prognosis of patients with acute paraquat poisoning based on three biomarkers of the blood test including the neutrophilic granulocyte ratio, leukocyte count, and eGFR(MDRD). The neutrophilic granulocyte ratio and leukocyte count reflect the immune status of the body. Several studies have shown that the immune system of patients was activated during the paraquat poisoning [9, 10]. As expected, the neutrophilic granulocyte ratio and leukocyte count increased in both survivors and nonsurvivors (Table 1). The activation of the immune system is a protective mechanism for the body in response to external stimuli. However, if the immune system is overactivated, the immune system may damage normal tissues and organs or even cause death [11]. In our study, we found that the neutrophilic granulocyte ratio and leukocyte count were significantly higher in the nonsurvivors compared to the survivors (Table 1 and Figures 1(a)–1(c)). Paraquat is mainly excreted through the kidney. Thus, the concentration of paraquat was pretty high in the kidneys of the patients with acute paraquat poisoning, and it causes remarkable acute renal injury in the early stage of poisoning [12]. The glomerular filtration rate (eGFR(MDRD)) is the best overall index of kidney function. In our study, we found that the eGFR(MDRD) significantly reduced in patients with acute paraquat poisoning at admission, which reflects that the paraquat could cause kidney damage upon intake. More interestingly, compared to the survivors, the eGFR(MDRD) was much lower in the nonsurvivors (Figure 3(f)).

The development and progression of acute paraquat poisoning was associated with systemic inflammation, especially the neutrophils and lymphocytes [13–15]. Several studies reported that the extensive influx of neutrophils was rapidly promoted and the apoptosis of neutrophils was significantly inhibited after paraquat ingestion, whereas the apoptosis of lymphocytes was accelerated by intracellular redox state imbalance [14, 15]. In this study, we found that high neutrophils and low lymphocytes were associated with the outcome of acute paraquat poisoning (Figures 2(a) and 2(b)). Since the neutrophil-to-lymphocyte ratio (NLR) reflected the systemic inflammatory and stress responses with the rise of neutrophils and apoptosis of lymphocytes, NLR had been proved to have the possibility to serve as an inflammatory biomarker in a variety of medical conditions, including major cardiac events [16], ischemic stroke [17], cerebral hemorrhage [17–19], cancers [20], sepsis and infectious pathologies [21], and COVID-19 infection [22, 23]. Recently, Cao et al. found that the NLR could be used as an early predictor of survival in patients with acute paraquat poisoning [15]. In this study, the NLR was also investigated to evaluate its diagnostic values for the prognosis of patients with acute paraquat poisoning. We found that the NLR of the nonsurvivors was significantly higher than that of the survivors and the AUC of the NLR to predict the mortality in patients with acute paraquat poisoning was 0.8667 (95% CI: 0.7277-1.006). The sensitivity and specificity were 71.43% and 93.33%.

There are some limitations to this study. For instance, we just include 29 patients in our study. The sample size is too small because the number of people poisoned by paraquat is relatively small due to strict government control. Thus, it needs expansion and multicenter research in further studies. Second, the concentration of paraquat in blood for most patients was too low to evaluate its diagnostic value.

## 5. Conclusion

In our study, we established a novel, simple, cheap, and effective method to evaluate the prognosis of patients with acute paraquat poisoning based on the combination of the neutrophilic granulocyte ratio, leukocyte count, and eGFR(MDRD).

## Figures and Tables

**Table 1:** Patient characteristics and clinical features of patients with acute paraquat poisoning.

Case no.	Dead (*n* = 15)	Alive (*n* = 14)
Age	46.2 ± 20.8	40.9 ± 15.6
Sex (female)	8 (53%)	8 (57%)
Time from poisoning to treatment (h)	16.3 ± 22.7	31.2 ± 33.1
Toxic dose (mL)	35.4 ± 17.9^∗^	19.3 ± 15.3
Blood routine at admission		
Neutrophilic granulocyte ratio (50-70%)	90.5 ± 3.2^∗∗∗^	74.7 ± 15.7
Lymphocyte ratio (20-40%)	5.5 ± 1.9^∗∗^	18.0 ± 14.2
Leukocyte count (4.0 ~ 10.0 × 10^9^/L)	17.4 ± 8.3^∗∗^	9.2 ± 4.0
Eosinophil count (0.05 ~ 0.30 × 10^9^/L)	0.007 ± 0.01	0.02 ± 0.03
Basophil count (0.12 ~ 0.8 × 10^9^/L)	0.01 ± 0.02	0.01 ± 0.01
Erythrocyte count (4.0 ~ 5.5 × 10^12^/L)	4.6 ± 0.7	4.5 ± 0.7
Hemoglobin (110~165 g/L)	141.8 ± 13.2	130.6 ± 15.4
Platelet count (100 ~ 300 × 10^9^/L)	172.2 ± 84.1	141.6 ± 42.0
Neutrophil-to-lymphocyte ratio (NLR)	18.6 ± 7.7^∗∗∗^	7.9 ± 6.8
Blood biochemistry at admission		
ALT (0~40 IU/L)	71.7 ± 120.5	25.7 ± 23.3
AST (0~45 IU/L)	108.7 ± 159.5	24.5 ± 14.8
TBIL (1.7~17.1 *μ*mol/L)	30 ± 34.7	15.4 ± 9.2
ALB (35~55 g/L)	44.8 ± 5.5^∗^	40.4 ± 3.5
BUN (1.8~7.1 mmol/L)	7.7 ± 3.8	5.5 ± 3.1
Crea (44~133 mmol/L)	134.3 ± 77.1^∗∗^	64.3 ± 35.8
Ca (2.2~2.7 mmol/L)	2.2 ± 0.2	2.2 ± 0.2
K (3.5~5.5 mmol/L)	3.9 ± 1.0	3.6 ± 0.3
Na (135~145 mmol/L)	139.5 ± 4.2	139.9 ± 2.7
CRP (0~50 mg/L)	25.4 ± 26.4	19.7 ± 24.8
eGFR(MDRD) (80-120 mL/min)	59.2 ± 56.4^∗∗^	134.9 ± 55.3
PCT (<0.1 ng/mL)	1.7 ± 2.9	0.08 ± 0.09

**Table 2:** Variables in the equation.

	*B*	S.E.	Wald	df	Sig.	Exp(*B*)
Neutrophilic granulocyte ratio	0.678	0.366	3.426	1	0.064	1.971
Leukocyte count	0.484	0.267	3.299	1	0.069	1.623
eGFR(MDRD)	-0.034	0.019	3.297	1	0.069	0.967
Constant	-62.54	33.227	3.543	1	0.06	0

## Data Availability

All data generated or analyzed during this study are included in this published article. Raw and processed data are available upon request.

## Supplementary Materials

Supplementary Materials Table s1: patient characteristics and clinical features of patients with acute paraquat poisoning.
