# Supplementary material for: Identify the Early Predictor of Mortality in Patients with Acute Paraquat Poisoning
Source: Biomed Res Int. 2020 Dec 31;2020:8894180. doi: 10.1155/2020/8894180 (PMC7790583; doi:10.1155/2020/8894180)
Supplement: Supplementary Materials — Table s1: patient characteristics and clinical features of patients with acute paraquat poisoning. [file 8894180.f1.pdf]

**Table s1. Patient characteristics and clinical features of patients with acute paraquat poisoning.**

| Case No. | Sex | Age | Time from poisoning to treatment (h) | Toxic dose(ml) | Survival |
|---|---|---|---|---|---|
| 1 | Female | 39 | 13 | 30ml (20%) | Dead |
| 2 | Female | 33 | 7 | 20ml | Dead |
| 3 | Female | 50 | 11 | 50ml | Dead |
| 4 | Female | 49 | 16 | 50ml | Dead |
| 5 | Female | 28 | 96 | 40ml | Dead |
| 6 | Female | 48 | 7 | 20ml | Dead |
| 7 | Female | 72 | 15 | 100ml (20%) | Dead |
| 8 | Female | 68 | 7 | 100ml (20%) | Dead |
| 9 | Male | 28 | 6 | 60ml | Dead |
| 10[a] | Male | 20 | 24 | UN | Dead |
| 11 | Male | 21 | 4 | 50ml | Dead |
| 12 | Male | 64 | 5 | 60ml | Dead |
| 13 | Male | 92 | 10 | 30ml | Dead |
| 14 | Male | 51 | 7 | 50ml | Dead |
| 15 | Male | 30 | 16 | 20ml | Dead |
| 16[b] | Female | 56 | 48 | UN | alive |
| 17 | Female | 24 | 8 | 50ml | alive |
| 18 | Female | 29 | 5 | 10ml | alive |
| 19 | Female | 33 | 3 | 3ml | alive |
| 20 | Female | 49 | 5 | 30ml | alive |
| 21 | Female | 49 | 72 | 30ml | alive |
| 22 | Female | 49 | 15 | 5ml | alive |
| 23 | Female | 52 | 16 | 5-10ml | alive |
| 24 | Male | 29 | 5 | 7.5ml | alive |
| 25[c] | Male | 71 | 72 | UN | alive |
| 26 | Male | 50 | 5 | 20ml | alive |
| 27[d] | Male | 25 | 96 | UN | alive |
| 28[e] | Male | 14 | 16 | UN | alive |
| 29 | Male | 43 | 72 | 30ml | alive |

**[a]:** unknown; **[b]:** Eating vegetables sprayed with Paraquat; **[c]:** skin contact during spray paraquat; **[d]:** Oral contact during spray paraquat; **[e]:** Eating fruit sprayed with Paraquat
